# Size-driven ferroelectric–paraelectric phase transition in TGS nanocomposites

**DOI:** 10.1007/s11051-013-1807-y

**Published:** 2013-07-03

**Authors:** A. Cizman, T. Antropova, I. Anfimova, I. Drozdova, E. Rysiakiewicz-Pasek, E. B. Radojewska, R. Poprawski

**Affiliations:** 1 Institute of Physics, Wrocław University of Technology, Wybrzeże St. Wyspiańskiego 27, 50-370 Wrocław, Poland; 2 Grebenshchikov Institute of Silicate Chemistry, Russian Academy of Sciences, Nab. Makarova, 2, Saint Petersburg, Russia

**Keywords:** Size effect, Porous matrix, Ferroelectric nanocomposites, Phase transition

## Abstract

Dielectric properties of porous glass nanocomposites with TGS crystals embedded into six porous matrices with average pore size from 5 to 312 nm were investigated in the temperature range from 280 to 380 K at selected frequencies. The results are discussed based on the effect of the particle size on the phase transition temperature of TGS nanocomposites. Temperature–size phase diagram of TGS composites was derived. Non-monotonic character of the temperature-driven phase transition (*T*_p_) with the decreasing particle size was determined. The nature of the *T*_p_ variation can be ascribed to the size-effect theoretically predicted by Zhong et al. (Phys Rev B 50:698–703, 1994).

## Introduction

Ferroelectric materials show a wide range of properties including piezoelectric, pyroelectric, electrooptic, and nonlinear optical properties that underlie the practical applications of these materials. The piezoelectric sensors, nonvolatile ferroelectric random-access memories (FeRAM), pyroelectric infrared detectors, micromachines, actuators, acoustic wave devices (sensors, resonators, filters), and electro-optical or photonic devices are typical examples of the applications. Striving for miniaturization of today’s devices raises the fundamental questions on the minimum size for ferroelectricity, on the influence of the ferroelectric particle size on their physical properties and the phase transition temperature. In order to answer this question the size-effect in ferroelectric materials has been examined in numerous theoretical and experimental studies (Zhong et al. 1994; Charnaya et al. 2007; Morozovska et al. 2010; Strukov et al. 2004; Cizman et al. 2012).

Different chemical methods including the compound thin film method, sol–gel or the porous glass method, have been used for the synthesis of ferroelectric nanoparticles (Rysiakiewicz-Pasek et al. 2000; Kumzerov and Vakhrushev 2004). The current glass manufacturing technology gives the possibility to produce porous matrix with controlled parameters: size and shape of pores. It should be pointed out that by controlling the glass manufacturing technology it is possible to obtain a porous glass with a pore size between 5 and 1000 nm with a very narrow pore size distribution. Recent research of ferroelectric materials embedded in porous glass matrices have shown that the new nanocomposites exhibit different properties than those of bulk and are attractive for application reasons (Strukov et al. 2004; Cizman et al. 2012). Further studies of the temperature dependence of the particle size are a necessary and important step toward an understanding of the size-effect problem. In the studies of size-effect in AHS and KDP nanocomposites, we recently have shown that the phase transition temperature depends on the particle size and changes non-monotonically with its decreasing size (Cizman et al. 2012). It is evident that ferroelectric materials which exhibit the phase transition above the room temperature are more attractive for industrial application. The triglycine sulfate (TGS) is one of the important ferroelectric materials which found various applications and fulfills the above condition. TGS crystals exhibit a typical second-order ferroelectric phase transition at temperature *T*_C_ = 322 K (Landolt-Bornstein 1982) from the rhombohedral system with *P2*_*1*_/m symmetry to the ferroelectric phase with the non-centrosymmetric monoclinic point group *P2*_*1*_. Dielectric properties of triglycine sulfate compressed powder with different grain sizes (i.e., 22–123 μm) were investigated by Ostrowski et al. (2012).

The Landau theory for ferroelectric crystals in the confined geometry was clearly described by Zhong et al. (1994), by taking the polarization gradient, the surface and energy density into account. In this model, authors introduced the surface extrapolation length *δ* meaning a distance from the crystallite surface at which the linear extrapolation of the polarization near the surface fell to zero. When the polarization at the surface (*P*_surf_) is reduced with respect to the crystal interior polarization (*P*_int_), then *δ* > 0, when the *P*_surf_ > *P*_int_, then *δ* < 0. It should be pointed out that the extrapolation length depends on a particle structure, its size, shape and an interaction between the particles (Zhong et al. 1994; Charnaya et al. 2007).

For the spherical ferroelectric particles belonging to the cubic system, the size dependence of the phase transition temperature (*T*_C_) can be approximately written as:

$$ T_{\text{C}} = T_{{{\text{C}}\infty }} - \frac{6D}{\delta Ad} $$

where *T*_C∞_—is the Curie temperature of the bulk crystals, *A* and *D* are the Landau expansion coefficients, *d* is the particle diameter. The extrapolation distance (*δ*) depends on the crystal structure and the particle size (a coordination number of particles inside the crystal and on the crystal surface).

The following conclusion can be drawn based on the phenomenological description:

1. For the ferroelectric particle with *δ*_∞_ > 0, (where the *δ*_∞_ denotes the extrapolation length of the bulk crystals) the phase transition temperature monotonically decreases with the decreasing particle size.
2. For the ferroelectrics with *δ*_∞_ < 0, the particle size reduction initially causes *T*_C_ to increase as compared to the bulk, whereas when the *δ* becomes positive, it results in a reduction of the phase transition temperature.
3. The size-effect does not affect the phase transition order.
4. Pore size distribution caused a smearing of the phase transition in porous glass ferroelectric nanocomposites.
5. If particle sizes are sufficiently small (less than the correlation distance) the ferroelectric ordering does not arise.

The aim of the study is to examine the size-effect on the physical properties and the phase transition in the case of TGS crystals embedded in porous matrices with a wide range of the mean pore diameters by the dielectric spectroscopic method.

## Experiment

A starting material for the preparation of porous glass was homogeneous sodium borosilicate (Na_2_O–B_2_O_3_–SiO_2_) glass (Rysiakiewicz-Pasek et al. 2000). A phase separation was carried out by heating the initial glass at high temperatures. The temperature and time of the thermal treatment of the initial glass affect the average pore size (Antropova 2008).

**Fig. 1:**
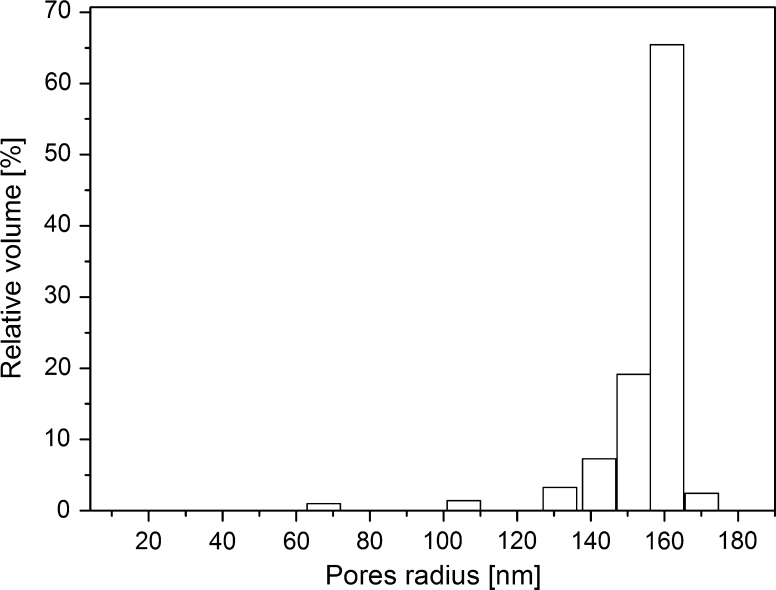
Pore size distributions for PG160 determined by the mercury intrusion porosimetry

For research purposes, after the phase separation, the glass plates were cut into dimensions of 10 × 10 × 0.5 mm^3^. Boron-rich phase was leached in hydrochloric acid solution. Silica gel remaining in the pores was removed by soaking the glass plates in potassium hydroxide solution. In order to obtain the final plates the porous glasses were washed in distilled water and dried at 400 K. The mercury porosimetry was used to determine the pore volume and the distribution of their sizes. The Washburn equation and a cylindrical pore model were used to calculate the pore diameter. In order to determine the specific surface area of pores the low temperature nitrogen sorption method was used. The example of pores size distributions of PG160 determined by the mercury intrusion porosimetry is shown in Fig. 1.

The structure parameters of porous glasses and their labels used in the text are shown in Table 1. The first column shows the signatures of porous glasses (number following the symbol PG represents the average pore radius in nm). In the next columns, the specific surface area and the porosity are presented. It is worth noting that the average pore sizes belong to the range from several to several hundreds of nm, and the specific surface areas of the pores vary from about 4 m^2^/g to about 140 m^2^/g.

**Table 1:** The structure parameters of the porous glasses

Glass	Mean pore radius *r* (nm)	Specific surface area *S* (m^2^/g)	Porosity *W* (%)
PG05	5	137.0	45
PG12	12	63.5	54
PG23	23	28.8	48
PG71	71	12.4	41
PG160	160	5.9	48
PG312	312	3.9	44

**Fig. 2:**
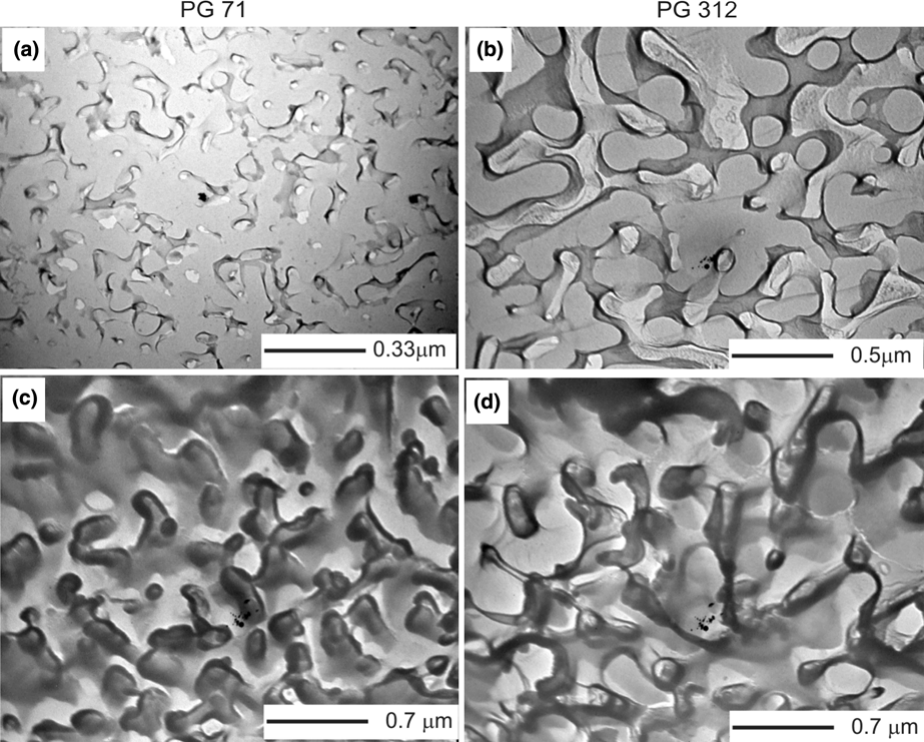
TEM photographs of the initial phase-separated glasses (**a**) and (**b**); and porous glasses obtained as a result of leaching them in HCl solution with the subsequent etching in KOH solution (**c**) and (**d**) for two different pore sizes with the average radius of 71 nm and of 312 nm, respectively

The transmission electron microscopy (TEM) study of the glasses was realized using an electronic microscope EM-125 at an accelerating voltage 75 kV with the resolution of 5 nm. In order to study the two-phase glasses, a method of platinum-carbon replica (Glauert 1972) was used. The replica was prepared from a freshly cleaved surface etched in either 2 % HF solution or in 2 % HCl solution at room temperature for 5–7 s. Figure 2 shows TEM images of the initial phase-separated glass and porous glasses.

TGS–PG nanocomposites were obtained by soaking of PG samples in TGS (99 % pure) saturated water solution at 340 K for 1 h. After this time the porous glass plates were dried at 380 K for 2 h. In order to enhance the filling factor, the above-mentioned process was repeated three times. In order to remove crystallites from the sample surface they were polished. The filling factor estimated by a weighting method was equal to about 85 %. For the removal of residual water the samples were dried before each experiment.

Investigation of dielectric properties was carried out on porous glass plates coated with silver paint electrodes. The measurements of the complex dielectric permittivity were done with the Novocontrol Alpha impedance analyzer, over the frequency range from 10 Hz to 10 MHz. Measurements were carried out in the range 280–360 K with the temperature rate of 1 K/min. The porous glasses obtained by the presented method served for a systematic study of the influence of the TGS particle sizes varying in a wide range on their physical properties and phase transitions.

## Results and discussion

**Fig. 3:**
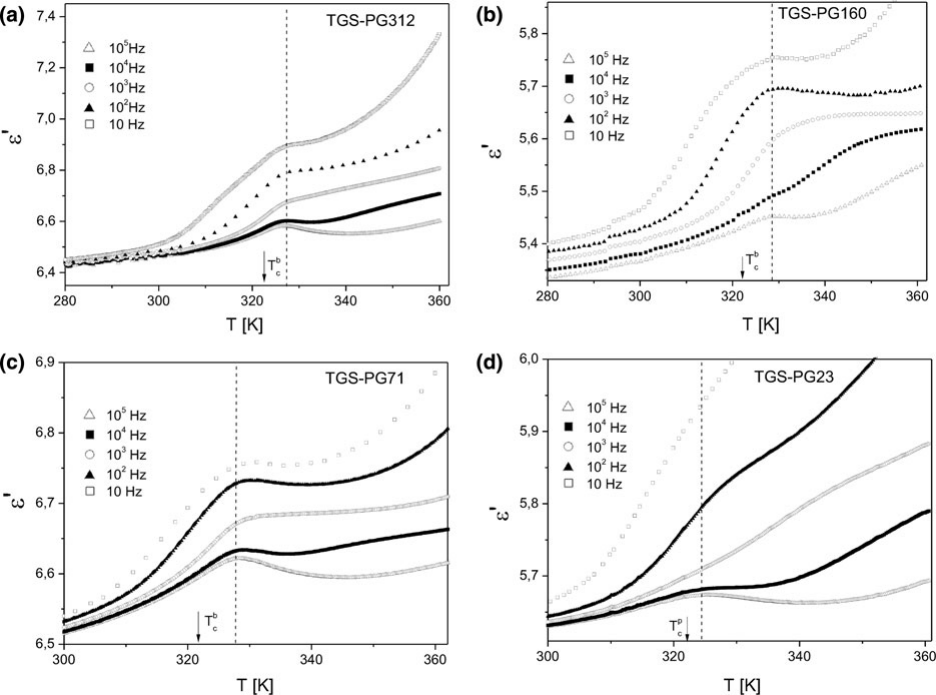
The temperature dependence of the real part of the dielectric permittivity *ε*’ of **a** TGS–PG312, **b** TGS–PG160, **c** TGS–PG71, and **d** TGS–PG23 at selected frequencies obtained on cooling

The results of the temperature dependence of the real part of the dielectric permittivity *ε*’ of TGS nanocomposites measured for several frequencies in the range of 10 Hz to 10 MHz are shown in Fig. 3.

Significantly, broader phase transition shape obtained for the confined TGS when compared to the bulk TGS crystals is related to the pore size distribution and the distribution of crystallites size. The different pore filling factor and the measurement uncertainty resulted in a scattering of the dielectric permittivity for investigated pore sizes.

For all TGS–porous glass nanocomposites the ferroelectric phase transition was indicated by a small but well-defined anomaly of the electric permittivity.

**Fig. 4:**
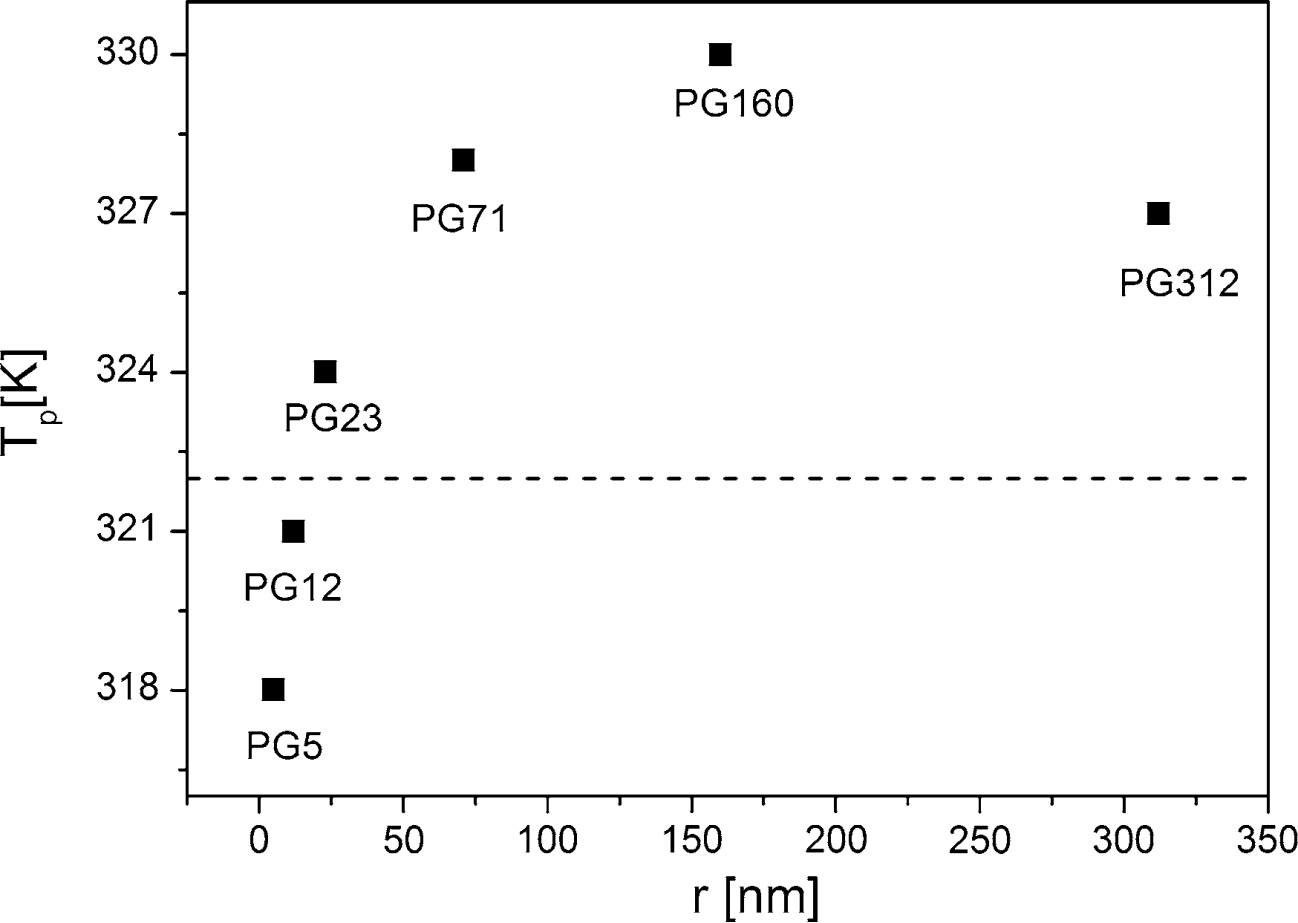
The phase transition temperature (*T*_C_) of TGS–PG nanocomposites (obtained as *ε*’_max_) as a function of the mean value of the pore radius (*r*). A *dash line* represents the phase transition temperature for a bulk TGS crystal

Based on the dielectric measurements the phase diagram representing the dependence of the phase transition temperature of the TGS nanocomposites on the mean value of the pore radius was derived. The phase transition temperature as a function of the TGS particle size embedded into porous matrices is shown in Fig. 4. The dashed line marks the phase transition temperature for bulk TGS crystals. The phase transition temperature of TGS nanocomposites was determined as a temperature of the maximum of the electric permittivity. For the TGS–PG12 and TGS–PG5, the maximum of the dielectric permittivity was not observed. In that case the phase transition temperature was calculated as the temperature of the inflection point of *ε*’(*T*). It is worth noting that at the examined frequency range from 10 Hz to 10 MHz the frequency rise leads to the decrease of *ε*’ below and above the phase transition.

The results showed that the phase transition temperature of TGS crystals embedded in porous matrices exhibited non-monotonic character, which was a characteristic feature of ferroelectrics with *δ* < 0.

## Conclusions

Dielectric measurements of TGS–porous glass nanocomposites with a mean value of the pore radius from 5 to 312 nm were carried out. An anomaly of the electric permittivity for TGS embedded into matrices of porous glasses (which proves the ferroelectric phase transition) was observed. The particle size dependence on the phase transition temperature was found.

The pore size dependence on the phase transition temperature was found to have a non-monotonic character which was typical for ferroelectrics with the surface layer polarization greater than the volume polarization. It should be pointed out that our results are the first experimental examination of the size-effect in TGS crystals performed in a wide range of particle sizes.
